# Tobacco Smoking Increases Immune Activation and Impairs T-Cell Function in HIV Infected Patients on Antiretrovirals: A Cross-Sectional Pilot Study

**DOI:** 10.1371/journal.pone.0097698

**Published:** 2014-05-19

**Authors:** Ranjini Valiathan, Maria J. Miguez, Bijal Patel, Kristopher L. Arheart, Deshratn Asthana

**Affiliations:** 1 Department of Pathology, University of Miami-Miller School of Medicine, Miami, Florida, United States of America; 2 Department of Psychiatry and Behavioral Science, University of Miami-Miller School of Medicine, Miami, Florida, United States of America; 3 Laboratory for Clinical and Biological Studies, University of Miami-Miller School of Medicine, Miami, Florida, United States of America; 4 School of Integrated Science and Humanities, Florida International University, Miami, Florida, United States of America; 5 Department of Public Health Sciences, Division of Biostatistics, University of Miami-Miller School of Medicine, Miami, Florida, United States of America; St. Jude Children's Research Hospital, United States of America

## Abstract

**Background:**

The influence of tobacco smoking on the immune system of HIV infected individuals is largely unknown. We investigated the impact of tobacco smoking on immune activation, microbial translocation, immune exhaustion and T-cell function in HIV infected individuals.

**Method:**

HIV infected smokers and non-smokers (n = 25 each) with documented viral suppression on combination antiretroviral therapy and HIV uninfected smokers and non-smokers (n = 15 each) were enrolled. Markers of immune activation (CD38 and HLA-DR) and immune exhaustion (PD1, Tim3 and CTLA4) were analyzed in peripheral blood mononuclear cells (PBMCs) by flow cytometry. Plasma markers of microbial translocation (soluble-CD14 - sCD14 and lipopolysaccharide - LPS) were measured. Antigen specific functions of CD4+ and CD8+ T-cells were measured, by flow cytometry, in PBMCs after 6 hours stimulation with Cytomegalovirus, Epstein-Barr virus and Influenza Virus (CEF) peptide pool.

**Results:**

Compared to non-smokers, smokers of HIV infected and uninfected groups showed significantly higher CD4+ and CD8+ T-cell activation with increased frequencies of CD38+HLA-DR+ cells with a higher magnitude in HIV infected smokers. Expressions of immune exhaustion markers (PD1, Tim3 and CTLA4) either alone or in combinations were significantly higher in smokers, especially on CD4+ T-cells. Compared to HIV uninfected non-smokers, microbial translocation (sCD14 and LPS) was higher in smokers of both groups and directly correlated with CD4+ and CD8+ T-cell activation. Antigen specific T-cell function showed significantly lower cytokine response of CD4+ and CD8+ T-cells to CEF peptide-pool stimulation in smokers of both HIV infected and uninfected groups.

**Conclusions:**

Our results suggest that smoking and HIV infection independently influence T-cell immune activation and function and together they present the worst immune profile. Since smoking is widespread among HIV infected individuals, studies are warranted to further evaluate the cumulative effect of smoking on impairment of the immune system and accelerated disease progression.

## Introduction

The prevalence of tobacco smoking among people living with HIV is as high as 70% [1]. Although combination antiretroviral therapy (cART) has resulted in enhanced immune reconstitution, the extent of improvements are quite variable making the effects of smoking on restoration of immune function difficult to elucidate. Notably, evidence indicates that despite years of successful treatment, immune activation (IA) and markers of inflammation remain abnormally high during HIV infection [2]. These findings are concerning given that IA has proven a better predictor of disease progression than plasma viral load (VL) [3]. Recent studies have highlighted the importance of gut microbial translocation (MT) as a major contributing factor for IA [4]–[6]. Levels of soluble CD14 (sCD14) and bacterial lipopolysaccharide (LPS), markers of MT [6], [7], remain high in HIV infected patients even after prolonged cART with viral suppression. During the course of HIV infection, T-cell functions such as proliferation and cytotoxic potential appear to diminish gradually leading to an incremental progression toward immune exhaustion (IE) [8]. Several markers of IE like Programmed death-1 (PD1), T-cell Ig domain and mucin domain-3 (Tim3), and Cytotoxic T-Lymphocyte Antigen-4 (CTLA4) are negative regulators of IA and are preferentially up-regulated on T-cells during HIV infection [9].

Cigarette smoke (CS) affects a wide range of immune functions impacting innate and adaptive host immunity [10], [11]. Clinical and experimental studies have been inconsistent which might be due to the nature of CS which has been shown to be both pro-inflammatory and immunosuppressive [12], [13]. Increased levels of pro-inflammatory cytokines have been reported in chronic smokers [14] as well as in HIV infected patients [15]. T-cells from smokers exhibit difference in proliferation response to T-cell mitogens and also in numbers, indicating defective T-cell responses [10], [16], [17]. Proteomics and transcriptomic studies also reveal that genes and proteins involved in immune function are perturbed by CS [18], [19]. Tobacco use has been known to significantly increase the risk of pulmonary diseases in HIV infected subjects along with many deleterious effects on antiretroviral treatment [20], [21].

Although it is known that HIV infection [3] and smoking [22] could impact T-cell activation, the effect of tobacco smoking on IA and other associated immune defects like MT or IE is not well understood in the context of HIV infection. We carried out this pilot study to investigate the effect of tobacco smoking on HIV infection. Here, we hypothesize that despite virologic suppression, the combination of smoking and HIV infection leads to chronic IA, thereby placing HIV-infected smokers at a higher risk of infection compared to HIV infected non-smokers and HIV uninfected age-matched controls. Our findings suggest that smoking in HIV infected people can lead to increased IA, MT and impairment of CD4+ and CD8+ T-cell functions that could eventually influence disease progression and management.

## Methods

### Ethics Statement

The study was approved by the University of Miami Institutional Review Board. Voluntary signed informed consent was obtained from every participant prior to enrollment in the study.

### Study Participants

The present study was conducted on four groups of participants: HIV infected smokers (HIV^+^S, n = 25), HIV infected non-smokers (HIV^+^NS, n = 25), HIV uninfected-smokers (HIV^neg^S, n = 15), and HIV uninfected non-smokers (HIV^neg^NS, n = 15). The HIV infected participants were referred to the study from local HIV providers and community clinics as well as HIV testing centers. All HIV infected participants in this study were on cART and virologically suppressed (VL<50 copies/ml) at the time of enrollment. Examination for each participant included the use of standardized research questionnaires regarding tobacco, alcohol, drug use, past and present medical history, physical examination and laboratory testing. Our participant recruitment was based on the National Health Interview Survey (NHIS) which defines current smokers as individuals who have smoked at least 100 cigarettes and now smoke either every day or some days of the week. Participants were enrolled if they were between 18–55 years of age and a current tobacco smoker. Any individual with a significant history of medical and immunological illness other than HIV and tobacco usage like liver cirrhosis, myopathies, pregnancy, malignancies, psychiatric conditions, malnutrition, cardiovascular diseases and immunosuppressive conditions were excluded. Drug abusers and daily hazardous alcohol abusers were also excluded from the study. The demographic characteristics of the four study groups are shown in Table 1.

**Table 1:** Characteristics of the study participants.

Parameter	HIV^+^S	HIV^+^NS	HIV^neg^S	HIV^neg^NS
**Number**	25	25	15	15
**Male**	15	10	9	8
**Age (years)**	46 (24–52)	46 (38–52)	41 (22–53)	39 (27–51)
**BMI (kg/m^2^)**	30 (20–45)	33 (19–40)	28 (19–37)	33 (24–46)
**Drinks/week**	28 (0–112)	8 (0−140)	10 (0–25)	1 (0–4)
**Cigarettes/day**	14 (2–30)	0	13 (1–28)	0
**WBC (cells/µl)**	5727 (2500–10800)	5963 (4000–11300)	n.a	7125 (3700–9600)
**Lymphocytes (%)**	38 (25–50)	38 (23–53)	n.a	37 (18–54)
**CD3 (%)**	79 (59–92)	77 (61–91)	70 (50–80)	74 (60–84)
**CD4 (%)**	31 (8–51)	27 (8–45)	44 (28–56)	44 (29–58)
**CD8 (%)**	43 (29–67)	46 (30–63)	25 (13–38)	33 (13–57)
**HIV viral load (RNA copies/ml)**	<50	<50	n.a	n.a

The table shows demographic characteristics of the study groups [mean (range)]. All HIV infected participants in this study were on cART and virologically suppressed (VL<50 copies/ml) at the time of enrollment. Participants were enrolled if they were between 18–55 years of age and a current tobacco smoker based on the survey questionnaire. Participants were excluded for any medical disorders and illicit drug use. n.a – not available.

### Processing of Blood Samples

Blood was drawn by venipuncture and collected into heparinized tubes for peripheral blood mononuclear cell (PBMC) isolation and in EDTA tubes for plasma collection. Samples were processed immediately after collection. Fresh PBMCs were isolated using standard density gradient centrifugation method using Ficoll Hypaque (GE Health Care Bio-sciences, PA). Cells at the interface containing PBMCs were collected and washed twice in phosphate-buffered saline (PBS; pH 7.2) and resuspended in RPMI 1640 (Cellgro, Mediatech, Inc. Manassas, VA) supplemented with 10% heat-inactivated fetal bovine serum, 100 U/ml penicillin and 100 µg/ml streptomycin. PBMC were cryopreserved at −140°C and plasma was aliquoted and stored at −80°C until ready to be assayed. Once thawed, the PBMCs were rested for 12 hours at 37°C, 5% CO2 and 100% humidity prior to using them for assays. Cell recovery was >80% and viability was >95% in all instances.

### Markers of Immune Activation and Exhaustion by Multicolor Flow Cytometry

Thawed PBMCs (1×10^6^ cells/ml) were rested overnight and stained with cell surface markers against CD3, CD4, CD8, CD38, HLA-DR, PD1, Tim3 and CTLA4 (Biolegend, San Diego, CA) at room temperature. Following incubation in the dark, cells were washed, re-suspended in PBS with 1% paraformaldehyde and acquired on FACSCanto Flow Cytometer (BD Bioscience, San Jose, CA) after proper instrument settings, calibration, and compensation [23], [24] and analyzed using Diva software (version 5.0). A minimum of 200,000 events were analyzed and gating was based on isotype controls. The CD4+ and CD8+ T-cells were gated from CD3+ T-cells and analyzed for IA (HLA-DR and CD38) and IE (PD1, CTLA4 and Tim 3).

### Antigen Specific T-cell Function by Intracellular Staining

For the evaluation of intracellular cytokine production, PBMCs (1×10^6^ cells/ml) were stimulated with 2 µg/ml Cytomegalovirus, Epstein - Barr virus and Influenza Virus (CEF-Peptide Pool, NIH AIDS Reagent Program). Staphylococcal Enterotoxin-B (SEB, 2 µg/ml) was included as a positive control and medium as negative control. Cultures were incubated in the presence of protein transport inhibitor, Brefeldin A (10 µg/mL; Sigma-Aldrich) and Monensin (0.7 µl/ml; E-biosciences). For the detection of degranulation, anti-CD107a was added at culture initiation together with the aforementioned stimuli and incubated at 37°C for 6 hours. After incubation, cells were washed and stained with Abs for surface markers- CD3, CD4 and CD8. Cells were then washed, fixed, permeabilized and stained intra-cellularly with IL-2 and IFN-γ mAbs, washed, re-suspended in PBS with 1% paraformaldehyde and acquired on FACSCanto Flow Cytometer and analyzed by Diva software as mentioned previously. Total CD4+ and CD8+ T-cells were analyzed for the degranulation marker, CD107a and cytokines (IL-2, IFN-γ).

### Markers of Microbial Translocation

Plasma levels of sCD14 and LPS were measured to detect microbial translocation.

### sCD14 Assay

Plasma levels of sCD14 were quantified [7], [25] using human sCD14 ELISA kit (R & D systems, Minneapolis, MN). Plasma (10 µl) was diluted 400-fold and assayed in duplicate per manufacturer’s recommendations. Results of sCD14 levels are expressed in ng/ml.

### LPS Assay

Levels of LPS were measured in plasma by using the limulus amebocyte lysate (LAL) chromogenic endpoint assay (Lonza Group Ltd, Allendale, New Jersey, USA) according to manufacturer’s recommendations. Plasma (10 µl) was diluted 5-fold in endotoxin-free water, and heat-inactivated at 80±5°C for 15 min to inactivate inhibitory plasma proteins before the assay. Following subtraction of the background levels, results were calculated relative to an *Escherichia coli* endotoxin standard provided with the assay [4] and results of LPS are expressed in pg/mL.

### Multiplex Cytokine Detection

Plasma levels of cytokines were measured using a customized MILLIPLEX Cytokine Human Ultrasensitive magnetic bead panel (EMD Millipore, Billerica, MA) following manufacturer’s instructions. Briefly, plasma samples were thawed, vortexed and centrifuged at 10,000 RPM for 5 min at 4°C prior to testing. Undiluted plasma was incubated over night with a mixture of beads specific for interleukins (IL-1β, IL-6, IL-8, IL-17) and tumor necrosis factor alpha (TNFα) at 4°C with shaking. After washing, the beads were incubated with biotinylated detection antibodies for 1 hour at room temperature. Streptavidin-PE was then added to the wells and allowed to incubate for 30 minutes at room temperature. The beads were then washed and diluted with 150 µl Sheath Fluid before acquisition on the MAGPIX instrument (Luminex Corporation, Austin, TX). The mean fluorescent intensity (MFI) data were analyzed with MILLIPLEX Analyst Software V.3.5 (EMD Millipore). Cytokine concentrations were determined based on standard curves and expressed in pg/ml.

### Statistical Analysis

Statistical analysis began with a multivariate analysis of variance to determine if there were significant group differences among the dependent variables. If the Wilks Lambda statistic test indicated that significant differences were present, analysis of covariance was performed on each independent variable, using a general linear model (GLM) approach. The first analysis of covariance model included groups (HIV^+^S, HIV^+^NS, HIV^neg^S and HIV^neg^NS) and covariates for a) number of alcoholic drinks per week and b) BMI. A second analysis was run for smokers only that included group (HIV^+^S and HIV^neg^S) with covariates for a) number of alcoholic drinks per week, b) BMI and c) number of cigarettes per day. One way ANOVA was used to perform five planned contrasts: HIV^+^S vs. HIV^+^NS, HIV^neg^S vs. HIV^neg^NS, HIV^+^S vs. HIV^neg^S, HIV^+^S vs. HIV^neg^NS and HIV^+^NS vs. HIV^neg^NS. Pearson correlation was used to assess the associations among variables, and linear regression was used to plot the data. Statistical test results with *P* values of ≤0.05 were considered statistically significant. One way ANOVA and Pearson correlation analyses were performed using Prism 6.02 (GraphPad, La Jolla, CA) and multivariate analysis using SAS 9.3 (SAS Institute, Inc., Cary, NC).

## Results

### Characteristics of Study Participants

The present study was conducted on four groups of participants: HIV^+^S, n = 25, HIV^+^NS, n = 25, HIV^neg^S, n = 15, HIV^neg^NS, n = 15. All HIV infected participants were receiving cART according to the standard of care for more than 6 months at the time of enrollment. Demographic characteristics of the study groups are shown in Table 1. All HIV infected participants in this study were virologically suppressed (VL<50 copies/ml). The white blood count (WBC), lymphocyte%, CD3%, CD4% and CD8% of the different study groups were not significantly different (Table 1). In addition, absolute numbers of CD3+, CD4+ or CD8+ T-cells were not significantly different between smokers and non-smokers in the HIV infected group (data not shown). Unfortunately, WBC and lymphocyte counts were unavailable for the HIV negative smokers.

### Immune Activation of CD4+ and CD8+ T-cells Increased in Smokers

**Figure 1:**
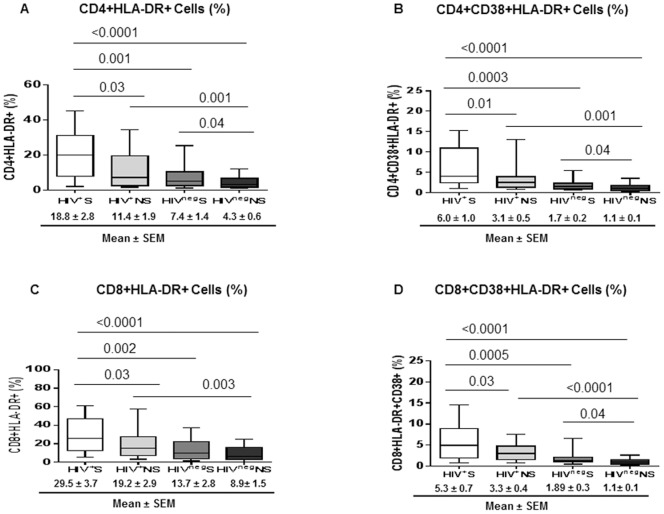
Higher cellular markers of T-cell activation in HIV infected and HIV uninfected smokers. Immune activation (CD38, HLA-DR) markers were evaluated by flow cytometry in CD4+ and CD8+ T-cells of HIV^+^S (n = 25), HIV^+^NS (n = 25), HIV^neg^S (n = 15) and HIV^neg^NS (n = 15). (A–D) Box plots show the frequency of activated T-cells: (A) CD4+HLA-DR+, (B) CD4+CD38+HLA-DR+, (C) CD8+HLA-DR+ and (D) CD8+CD38+HLA-DR+. Box plots represent median with 25th and 75th percentile borders, error bars represent 10th and 90th percentile. The mean ± standard error of mean (SEM) for each group are given below the bar for that particular group.

In adults with chronic HIV infection, markers of T-cell activation are reported to be elevated even after antiretroviral therapy [26]. We evaluated the phenotypic markers of T-cell activation (CD38 and HLA-DR) in our study groups. As shown in Fig. 1, frequencies of CD4+ and CD8+ T-cells expressing HLA-DR either alone or in combination with CD38 were highest in the HIV^+^S group compared to all other groups.

Frequencies of HLA-DR+ and CD38+HLA-DR+ CD4+ T-cells were significantly higher in HIV^neg^S compared to HIV^neg^NS (*P* = 0.04) which indicate the effect of smoking on CD4+ T-cell activation in the general population. The differences in frequency of CD4+ T-cell IA markers between the HIV^+^S and HIV^+^NS were also significantly higher (*P* = 0.03 and *P* = 0.01) indicating the synergistic effect of smoking on CD4 IA in HIV infected individuals. Additionally, HIV^+^NS demonstrated significantly higher frequencies of CD4+HLA-DR+ and CD4+CD38+HLA-DR+ cells compared to HIV^neg^NS (*P* = 0.001) indicative of the effect of HIV infection on CD4+ T-cell activation (Fig. 1A and 1B). The median fluorescence intensity (MFI) of HLA-DR and CD38 on CD4+ T-cells was also higher in HIV^+^S (data not shown) suggesting per cell based increase in CD4 IA in HIV infected smokers. The analysis of covariates for BMI and alcohol consumption/week in all four study groups showed a negative association between BMI and CD4 IA (HLA-DR+CD38+) (*P* = 0.03) but not with alcoholic drinks/week (Table 2). However, number cigarettes/day did not show any association with markers of CD4 IA in smoker groups. Similarly, the analysis of CD8+ T-cells showed higher levels of IA as measured by increased frequencies of CD8+HLA-DR+ and CD8+HLA-DR+CD38+ T-cells in HIV^+^S compared to HIV^+^NS indicating the combined effect of HIV and smoking on CD8+ T-cell activation (Fig. 1C and 1D). CD8+ T-cell IA was also evident in HIV^neg^S compared to HIV^neg^NS suggesting the impact of smoking on T-cell activation in the general population (Fig. 1D). Unlike CD4+ T-cell IA, CD8+ T-cell IA did not show any association with covariates BMI and alcohol consumption/week or number of cigarettes/week in the study groups (Table 2).

**Table 2:** Association of BMI, alcohol drinks/week and number of cigarettes/day with immune activation, microbial translocation and immune exhaustion.

Parameter	BMI	Drinks/week	Cigarettes/day
**CD4+HLA-DR+CD38+ (%)**	0.03*	0.66	0.64
**CD8+HLA-DR+CD38+ (%)**	0.31	0.1	0.5
**Plasma LPS (pg/ml)**	0.34	0.08	0.1
**Plasma sCD14 (ng/ml)**	0.18	0.95	0.63
**CD4+PD1+ (%)**	0.38	0.04*	0.19
**CD8+ PD1+ (%)**	0.55	0.08	0.99

We performed multivariate analysis to determine any significant group differences among the dependent variables. The first analysis model included groups (HIV^+^S, HIV^+^NS, HIV^neg^S and HIV^neg^NS) and covariates for a) number of alcoholic drinks/week and b) BMI. A second analysis was performed for smokers only that included groups (HIV^+^S and HIV^neg^S) with covariates for a) number of cigarettes/day, b) BMI and c) number of alcoholic drinks/week. The *P* values for the covariate regression parameters for each specific marker are shown in the table. * - Negative association.

T-cells produce various cytokines upon activation; therefore we evaluated the plasma levels of pro-inflammatory cytokines in the study participants. Although differences between smoking and non-smoking participants were not significant, increased levels of circulating IL-1β, IL-6, IL-8, IL-17 and TNF-a were observed in the smokers of both groups compared to the corresponding non-smoking group (Table 3).

**Table 3:** Plasma cytokine levels in the four groups of participants.

Cytokines (pg/ml)	HIV^+^S (n = 10)	HIV^+^NS (n = 10)	P value	HIV^neg^S (n = 10)	HIV^neg^NS (n = 7)	P value
**IL-1β**	3.128±2.08	0.86±0.33	0.40	3.62±1.33	3.57±1.66	0.98
**IL-6**	1.34±0.55	0.43±0.03	0.22	1.91±0.76	1.18±0.57	0.48
**IL-8**	10.00±3.54	4.09±1.58	0.48	9.08±1.41	5.81±1.75	0.16
**IL-17**	11.48±9.7	1.31±0.45	0.22	2.47±1.96	0.52±0.21	0.40
**TNF-α**	13.22±3.51	10.33±2.17	0.55	8.81±1.18	6.33±0.88	0.13

Circulating levels of cytokines were measured in the plasma of the four groups of participants using a customized MILLIPLEX Cytokine Human Ultrasensitive magnetic bead panel (EMD Millipore). The differences between smoking and nonsmoking participants were not significant. But increased levels of circulating IL-1β, IL-6, IL-8, IL-17 and TNF-α were observed in the smokers of both groups compared to the corresponding non-smoking group.

### Higher Levels of Microbial Translocation in Smokers

**Figure 2:**
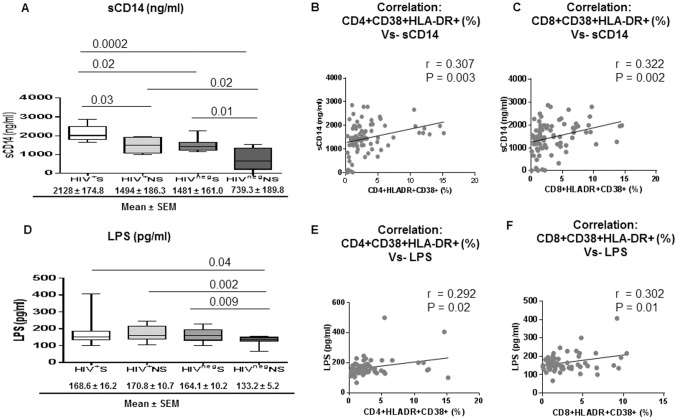
Elevated levels of microbial products in bloodstream of smokers are associated with immune activation. Plasma levels of LPS (Limulus amebocyte lysate chromogenic endpoint assay) and sCD14 (ELISA) were measured in HIV^+^S (n = 25), HIV^+^NS (n = 25), HIV^neg^S (n = 15) and HIV^neg^NS (n = 15). (A, B, C) Graphs depict the levels of sCD14 (ng/ml) in the 4 groups, HIV^+^S, HIV^+^NS, HIV^neg^S and HIV^neg^NS (A) and correlation of sCD14 levels with CD4+CD38+HLA-DR+ (%) (B) and CD8+CD38+HLA-DR+ (%) (C); (D, E, F) Graphs shows the levels of LPS (pg/ml) in the 4 groups, HIV^+^S, HIV^+^NS, HIV^neg^S and HIV^neg^NS (D) and correlation of LPS levels with CD4+CD38+HLA-DR+ (%) (E) and CD8+CD38+HLA-DR+ (%) (F). Box plots represent median with 25th and 75th percentile borders, error bars represent 10th and 90th percentile. Correlation between the two variables is indicated by the black continuous line in the correlation figures. The mean ± SEM for each group are given below the bar for that particular group.

Gut microbial translocation is known as an important contributor to IA in HIV infected patients [4], [27]. We analyzed the plasma levels of sCD14 and bacterial LPS, two reliable markers of microbial translocation, in the study groups. Plasma sCD14 is an indicator of LPS induced monocyte activation and has been recently reported as an important correlate in HIV disease progression and mortality [7]. Plasma sCD14 levels were significantly higher in smokers of both HIV infected (*P* = 0.03) and uninfected groups (*P* = 0.01) compared to their respective non-smoker groups (Fig. 2A). In addition, we found significantly higher levels of sCD14 in plasma from HIV^+^S compared to HIV^neg^S (*P* = 0.02) and in HIV^+^NS than HIV^neg^NS (*P* = 0.02, Fig. 2A). Importantly, correlation analysis showed that the sCD14 levels correlated directly with both CD4+ (*r* = 0.307; *P* = 0.003) and CD8+ T-cells IA (*r* = 0.322; *P* = 0.002) (Fig. 2B and 2C).

Circulating LPS is an indicator of translocation of microbial products from the gut into the bloodstream. Interestingly, plasma LPS levels were significantly higher in HIV^neg^S compared to HIV^neg^NS (*P* = 0.009) which further demonstrates the effect of smoking on microbial translocation regardless of HIV infection (Fig. 2D). Significantly higher levels of plasma LPS were noted in HIV^+^NS than HIV^neg^NS (*P* = 0.002; Fig. 2D) which could be HIV induced effect on gut permeability as described previously [4]. Additionally, plasma levels of LPS correlated directly with CD4+ (*r* = 0.292; *P* = 0.02) and CD8+ T-cell IA (*r* = 0.302; *P* = 0.01) (Fig. 2E and 2F). However, we did not observe any association between number cigarettes smoked/day, BMI or alcohol drinks/week with plasma levels of sCD14 or LPS (Table 2).

### Increased Levels of CD4+ T-cells Immune Exhaustion in Smokers

Markers of IE were analyzed in order to understand the relationship between IA and IE and its association with smoking in HIV infected individuals. Compared to non-smokers, smokers of both HIV infected and uninfected groups showed a significant increase in the frequencies of CD4+ T-cells expressing PD1, Tim3 and CTLA4, either alone or in combination.

**Figure 3:**
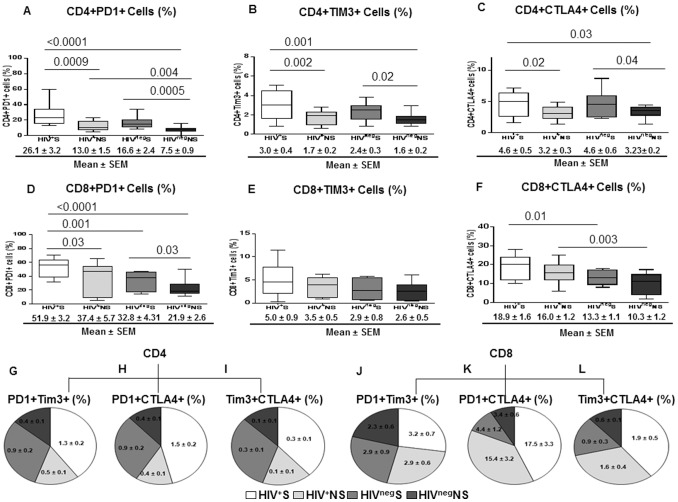
Higher levels of immune exhaustion markers in smokers compared to non-smokers. T-cell exhaustion markers (PD1, Tim3, and CTLA4) were evaluated by flow cytometry in CD4+ and CD8+ T-cells of HIV^+^S (n = 25), HIV^+^NS (n = 25), HIV^neg^S (n = 15) and HIV^neg^NS (n = 15). (A, B, C) frequency of exhausted CD4+ T-cells: CD4+PD1+ (A), CD4+Tim3+ (B), CD4+CTLA4+ (C) and (D, E, F) frequency of exhausted CD8+ T-cells: CD8+PD1+ (D), CD8+Tim3+ (E) and CD8+CTLA4+ (F). (G–L) Pie chart shows the comparison of frequencies of dual positive markers in CD4+ and CD8+ T-cells of HIV^+^S, HIV^+^NS, HIV^neg^S and HIV^neg^NS: CD4+PD1+Tim3+ (G); CD4+PD1+CTLA4+ (H); CD4+Tim3+CTLA4+ (I); CD8+PD1+Tim3+ (J); CD8+PD1+CTLA4+ (K) and CD8+Tim3+CTLA4+ (L). Box plots represent median with 25th and 75th percentile borders, error bars represent 10th and 90th percentile. The mean ± SEM for each group are given below the bar for that particular group.

Frequencies of CD4+PD1+ cells were higher in HIV^+^S compared to HIV^+^NS (*P* = 0.0009). HIV^neg^S also showed higher frequencies of CD4+PD1+ cells than HIV^neg^NS (*P* = 0.0005) indicating the influence of smoking on PD1 expression on CD4+ T-cells (Fig. 3A). In addition, CD4+PD1+ cells frequency was higher in HIV^+^NS than HIV^neg^NS (*P* = 0.004) showing the independent effect of HIV infection on PD1 levels. Frequencies of CD4+Tim3+ and CD4+CTLA4+ T-cells also showed a similar pattern to CD4+PD1+ T-cells in all the groups (Fig. 3B and 3C) indicting effect of tobacco on inducing multiple negative regulatory molecules on CD4+ T-cells and subsequent increase in immune exhaustion. Analysis of markers of immune exhaustion on CD8+ T-cells showed an increase in the frequencies of CD8+PD1+ cells in HIV^+^S when compared to HIV^+^NS (*P* = 0.03) and HIV^neg^S (*P* = 0.001) (Fig. 3D). Also, significantly higher frequencies of CD8+PD1+ T-cells were noticed in HIV^neg^S compared to HIV^neg^NS (*P* = 0.03) (Fig. 3D). In contrast to CD4+ T-cells, frequencies of CD8+Tim3+ and CD8+CTLA4+ T-cells were not significantly different between the groups (Fig. 3E and 3F).

In addition to the single expression pattern, we also analyzed the combined expression of markers of immune exhaustion on CD4+ and CD8+ T-cells. We analyzed the dual expression of PD1+Tim3+, PD1+CTLA4+, and Tim3+CTLA4+ on CD4+ and CD8+ T-cells. Our data showed that all these combinations were significantly higher on CD4+ T-cells of HIV^+^S compared to HIV^+^NS (*P* = 0.001; *P*<0.0001 and *P* = 0.005) and HIV^neg^NS (*P* = 0.001; *P*<0.0001and *P* = 0.0001) (Fig. 3G, 3H and 3I) indicating a significant impact of smoking on T-cell immune exhaustion in HIV infection. The HIV^neg^S also demonstrated higher expression of these combinations of inhibitory molecules (PD1+Tim3+, PD1+CTLA4+, and Tim3+CTLA4+) on their CD4+ T-cells suggesting a direct effect of smoking on CD4+ T-cell immune exhaustion in otherwise healthy individuals (Fig. 3G, 3H and 3I). Frequencies of CD8+ T-cells with combinations of PD1+CTLA4+ and Tim3+CTLA4+ cells did not show any significant difference between HIV^+^S and HIV^+^NS or HIV^neg^S which further confirm the selective effect of smoking on CD4+ T-cell exhaustion through the induction multiple inhibitory molecules. Taken together, the frequencies of CD4+ and CD8+ T-cells with increased expression of inhibitory molecules in combination was highest in HIV^+^S compared with other groups (Fig. 3G to 3L). Overall, our data suggest a more pronounced and selective effect of smoking on exhaustion of CD4+ T-cells than CD8+ T-cells as indicated by higher expression of multiple inhibitory molecules on CD4+ T-cells.

### Impaired Function of CD4+ and CD8+ T-cells in Smokers

**Figure 4:**
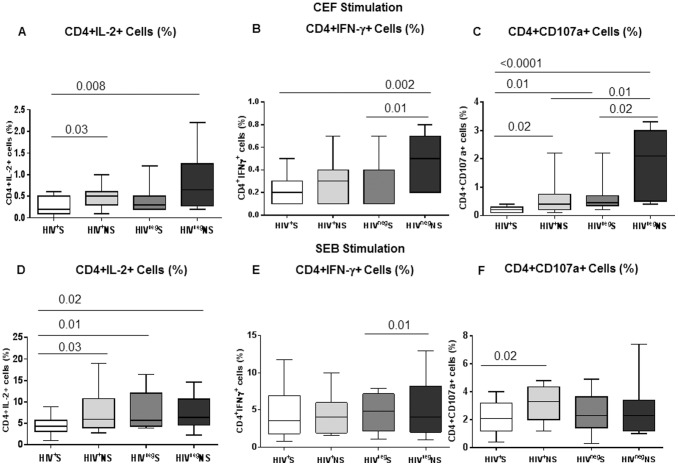
Decreased frequency of IL-2 and CD107a producing CD4+ T-cells in smokers compared to non-smokers. CD4+ T-cell function was analyzed by intracellular staining for cytokines IL-2, IFN-γ and degranulation marker CD107a using flow cytometry in the PBMCs of all four groups of participants [HIV^+^S (n = 25), HIV^+^NS (n = 25), HIV^neg^S (n = 15) and HIV^neg^NS (n = 15)] after 6hrs of stimulation with CEF and SEB in the presence of Brefeldin A and Monensin. (A, B, C) Box plots show the frequencies of CD4+ T-cells which produced IL-2 (A), IFN-γ (B) and CD107a (C) after stimulation with CEF in the four groups of participants. (D, E, F) Box plots show the frequencies of CD4+ T-cells which produced IL-2 (D), IFN-γ (E) and CD107a (F) after stimulation with SEB in the four groups of participants. Box plots represent median with 25th and 75th percentile borders, error bars represent 10th and 90th percentile.

In order to analyze the impact of smoking on antigen specific and polyclonal T-cell function of HIV infected and uninfected smokers, we stimulated PBMCs with CEF peptide pool and SEB. Upon CEF stimulation, frequencies of IL-2 and CD107a positive CD4+ T-cells were significantly lower in HIV^+^S (IL-2, *P* = 0.03; CD107a, *P* = 0.02, Fig. 4A and 4C) compared to HIV^+^NS. Additionally, frequencies of IFN-γ and CD107a positive CD4+ T-cells were significantly lower in HIV^neg^S (IFN-γ, *P* = 0.01; CD107a, *P = *0.02) compared to HIV^neg^NS (Fig. 4B and 4C). The significant decrease in the production of CD107a in HIV^+^NS compared to HIV^neg^NS (*P* = 0.01) indicate the effect of HIV infection on lowering the degranulation properties of antigen specific memory of CD4+ T-cells. After polyclonal stimulation with SEB, significantly lower IL-2 and CD107a expression was observed in CD4+ T-cells of HIV infected smokers (Fig. 4D and 4F). These results indicate a generalized defect in antigen specific and polyclonal CD4+ T-cell function in smokers and this could be the result of higher levels immune exhaustion of CD4+ T-cells in smokers.

**Figure 5:**
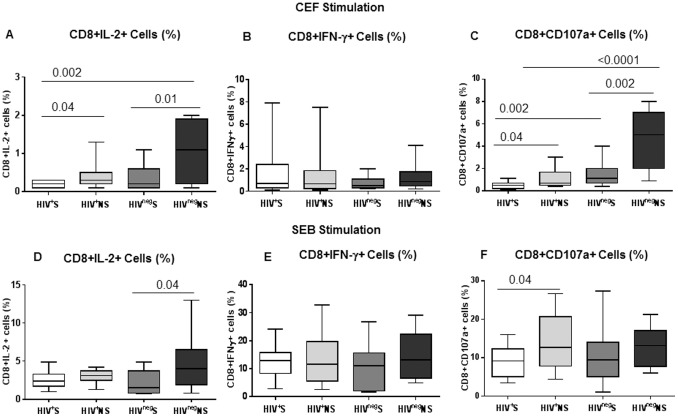
Lower frequency of IL-2 and CD107a producing CD8+ T-cells in smokers compared to non-smokers. CD8+ T-cell function was analyzed by intracellular staining for cytokines IL-2, IFN-γ and degranulation marker CD107a using flow cytometry in the PBMCs of all four groups of participants [HIV^+^S (n = 25), HIV^+^NS (n = 25), HIV^neg^S (n = 15) and HIV^neg^NS (n = 15)] after 6hrs of stimulation with CEF and SEB in the presence of Brefeldin A and Monensin. (A, B, C) Box plots show the frequencies of CD8+ T-cells which produced IL-2 (A), IFN-γ (B) and CD107a (C) after stimulation with CEF in the four groups of participants. (D, E, F) Box plots show the frequencies of CD8+ T-cells which produced IL-2 (D), IFN-γ (E) and CD107a (F) after stimulation with SEB in the four groups of participants. Box plots represent median with 25th and 75th percentile borders, error bars represent 10th and 90th percentile.

When CD8+ T-cell function was analyzed, a significantly lower production of antigen specific IL-2 and CD107a were observed in CD8+ T-cells of HIV^+^S and HIV^neg^S indicating the independent effect of smoking on CD8+ T-cell function (Fig. 5A, 5C). It is important to note that the antigen specific degranulation property of CD8+ T-cells was significantly affected by smoking even in HIV uninfected participants. Additionally, after polyclonal stimulation, significantly lower expression of CD107a was observed in HIV^+^S than HIV^+^NS and of IL-2 in HIV^neg^S than HIV^neg^NS. Our data indicate that like CD4+ T-cells, antigen specific CD8+ T-cell function is also significantly affected by smoking. The IFN-γ production by CD8+ T-cells after antigen and polyclonal stimulation was not affected by smoking in HIV infected or uninfected participants (Fig. 5B and 5E).

## Discussion

Persistent IA and inflammation associated with HIV infection accelerates the process of immunosenescence, likely placing HIV infected individuals at higher risk of developing infections. We demonstrate that HIV infected individuals who smoke tobacco products exhibit an increased state of immune activation and exhaustion compared to HIV infected non-smokers and HIV uninfected individuals. An increased level of sCD14 is indicative of monocyte activation and T-cell activation is implied by the increased surface expression of CD38 and HLA-DR. Higher T-cell activation was accompanied by an increase in exhaustion markers in HIV infected smokers and we also found increased levels of MT that correlated with markers of immune activation. These data represent the first comprehensive analysis of the combined effect of HIV infection and smoking on the status of the immune system in virologically controlled HIV infected smokers who are on cART. Moreover, the multivariate analyses indicate that being a member of the HIV positive smoking group was significantly associated with higher frequencies of CD4+CD38+HLA-DR+ (P = 0.0001), CD8+CD38+HLA-DR+ (P<0.0001) and CD4+PD1+ (P<0.0001) T-cells and higher levels of LPS (P = 0.002) and sCD14 (P<0.0001) compared to healthy controls (HIV^neg^NS) (data not shown). These findings are significant because they occur in a group of patients who are HIV infected smokers and are at higher risk for infections. Although it is reported that alcohol may work synergistically with HIV to promote microbial translocation and immune activation [28], our analysis did not show any significant effect of alcohol consumption or number of cigarettes smoked/day on immune activation and microbial translocation.

HIV infection results in chronic IA and overall immunological dysfunction, and both of these closely associate with the progression to AIDS [3], [26], [29]. Furthermore, even in those who are successfully treated with HAART, persistent IA is associated with increased morbidity and mortality [30]–[32]. Microbial translocation is one of the most important [4], [5] factors implicated in triggering IA in HIV-infected patients. The presence of bacteria-derived products in the bloodstream [4], [27] is sensed by toll-like receptors [33] that are responsible for inducing the production of inflammatory mediators that contribute to systemic IA. Microbial translocation has been associated with severity of HIV infection [34] and elevated levels of LPS in the plasma of HIV infected ART-treated individuals have been used as an indicator of long-lasting damage to the gut [27]. Even though VL of HIV^+^S and HIV^+^NS were controlled, smokers showed significantly higher CD4+ and CD8+ T-cell activation compared to non-smokers. HIV infected smokers also demonstrated increased levels of pro-inflammatory cytokines compared to rest of the groups [14], [15]. It is reported that under well monitored HIV care, HIV^+^S lose more life-years to smoking than to HIV [35]. An interesting observation was the higher CD4+ T-cell immune activation in HIV^neg^S than HIV^neg^NS indicating a direct association of smoking in the activation of CD4+ T-cells which could affect overall immune response. It is reported that levels of lipopolysaccharide-binding protein and sCD14 in the bronchoalveolar lavage of smokers are significantly higher than non-smokers [36]. Our analysis of gut MT showed higher levels of sCD14 and LPS in smokers and it correlated with CD4+ and CD8+ immune activation supporting the notion that MT might have a role in inducing IA [4], [26]. Increases in sCD14 levels in HIV^neg^S indicate the direct association of smoking with sCD14 levels. It is possible that gut permeability is damaged in smokers compared to non-smokers which might lead to MT and subsequent systemic IA. Our results indicate that MT might be the main factor behind IA in smokers and persistent IA is associated with accelerated disease progression in HIV infected individuals [37].

Our data indicate that there is selective up-regulation of multiple inhibitory molecules on CD4+ T-cells which might be significant contributor to the IE state and that CD4+ and CD8+ T-cells show different patterns of expression of inhibitory molecules in smokers [38]. In agreement with previous reports, we found increased expression of PD1 on both CD4+ and CD8+ T-cells of HIV infected subjects [39]. Additionally, significantly higher PD1 expression was observed in HIV^+^S than HIV^neg^S which indicates a synergistic effect of smoking with HIV on IE status of HIV infected individuals. Frequencies of other IE markers (Tim3 and CTLA4) along with combined IE marker frequencies (PD1 and Tim3, PD1 and CTLA4 and Tim3 and CTLA4) were also significantly higher in CD4+ T-cells of smokers of both HIV infected and uninfected groups. As we enrolled patients with undetectable VL in both smoking and non-smoking groups of HIV patients, viremia might not be the reason behind the observed difference in IE. The T-cells from smokers were more exhausted than non-smokers indicating that mechanisms other than viral persistence might be the reason for development of IE during HIV infection.

Although smoking independently enhanced T-cell activation in HIV^neg^S, data on the effects of smoking on immune function in HIV infected individuals are limited. Since increased expression of inhibitory molecules was observed on CD4+ and CD8+ T-cells of HIV^+^S and HIV^neg^S, we further analyzed the effect of smoking on the functionality of T-cells. We found significant differences in the IL-2 and CD107a production to recall responses and polyclonal stimulation in both CD4+ and CD8+ T-cells of smokers irrespective of their HIV status. These findings need further investigation as the reduced T-cell function in smokers will have long lasting effects on overall immune response to vaccinations or other infections. Reduced IL-2 production by CD4+ T-cells has been associated with decreased antibody responses to influenza vaccination in older people [40], [41]. The quality of CD4+ T-cells in smokers has been significantly affected and this defect in their immune function could explain why smokers develop serious complications during an infectious disease. Cigarette smoking is reported as an independent risk factor for invasive pneumococcal disease in immune-competent adults and this risk is positively correlated with smoking pack-years [42], which could be a result of smoking-associated reductions in T-cell function. In our study, specific T-cell functional capacity was reduced in smokers regardless of their HIV status and this is in agreement with previous proteomic and transcriptomic studies which reported that proteins and genes involved in immune-function are altered in smokers [18], [19]. HIV infected smokers are at increased risk of incidence of bronchitis [43] and bacterial pneumonia [44], [45] due to higher immune defects. Limitations of the study include the small sample size and absence of absolute counts for the HIV uninfected smokers.

As a pilot, however, this study demonstrates that HIV-infected ART-treated smokers who are virologically suppressed have higher levels of immune activation and exhaustion than HIV-infected age matched non-smokers and HIV uninfected controls. In conclusion, to the best of our knowledge, this is the first report on the effect of smoking on different immune markers in smokers, especially in an HIV infected cohort. Our study also provides evidence that smoking is associated with compromised immune cell functionality. Still there are many questions which need to be answered including whether IA could lead to opportunistic infections, whether there is accelerated replication of the virus, or whether there is increased damage to the immune system of smokers. Future studies should be focused on understanding the mechanisms of IA/IE and its impact on disease progression and its correlation with clinical outcomes in HIV-infected smokers. As recently suggested, viral persistence is facilitated by IA, and strategies to contain IA including control of MT need to be further explored in these patients.
